# FGD5-AS1 facilitates the osteogenic differentiation of human bone marrow-derived mesenchymal stem cells via targeting the miR-506-3p/BMP7 axis

**DOI:** 10.1186/s13018-021-02694-x

**Published:** 2021-11-12

**Authors:** Jun Li, Xingbiao Wu, Yaohua Shi, Hong Zhao

**Affiliations:** Department of Spinal Surgery, Changzhou Hospital of Traditional Chinese Medicine, No. 25 Heping North Road, Changzhou, Jiangsu 213000 P.R. China

**Keywords:** FGD5-AS1, miR-506-3p, BMP7, Osteoporosis, BMSC

## Abstract

**Background:**

Osteoporosis is a systemic disease characterized by impaired bone formation, increased bone resorption, and brittle bone fractures. The osteogenic differentiation of human bone marrow-derived mesenchymal stem cells (hBMSCs) is considered to be a vital process for bone formation. Numerous studies have reported that long non-coding RNAs (lncRNAs) are involved in the osteogenic differentiation of hBMSCs. The present study aimed to investigate the effect of FGD5 antisense RNA 1 (FGD5-AS1) on osteogenic differentiation.

**Methods:**

RT-qPCR was performed to detect the expression of FGD5-AS1, miR-506-3p, and osteogenesis-related genes OCN, OPN, OSX, and RUNX2. Western blotting was carried out to detect the protein levels of osteogenesis-related markers. In addition, the regulatory effect of FGD5-AS1 on osteogenic differentiation was detected through alkaline phosphatase (ALP) activity, Alizarin Red S (ARS) staining, and Cell Counting Kit-8 (CCK-8). Bioinformatics analysis and luciferase reporter assay were used to predict and validate the interaction between FGD5-AS1 and miR-506-3p as well as miR-506-3p and bone morphogenetic protein 7 (BMP7).

**Results:**

The RT-qPCR analysis revealed that FGD5-AS1 was upregulated in hBMSCs following induction of osteogenic differentiation. In addition, FGD5-AS1 knockdown attenuated hBMSC viability and osteogenic differentiation. Bioinformatics analysis and luciferase reporter assays verified that FGD5-AS1 could directly interact with microRNA (miR)-506-3p. Furthermore, miR-506-3p could directly target the 3′-untranslated region (3′-UTR) of BMP7. Additionally, functional assays demonstrated that miR-506-3p silencing could restore the suppressive effect of FGD5-AS1 knockdown on osteogenic differentiation and viability of hBMSCs, and miR-506-3p could attenuate osteogenic differentiation via targeting BMP7.

**Conclusions:**

Taken together, the results of the present study suggested that FGD5-AS1 could positively regulate the osteogenic differentiation of hBMSCs via targeting the miR-506-3p/BMP7 axis.

## Introduction

Osteoporosis is a chronic inflammatory bone disease characterized by the destruction of the bone trabecular structure and reduced bone density [1]. Due to the aging population, the incidence of osteoporosis in the elderly population is increasing [2], posing a major socioeconomic burden. Previous studies have shown that osteoporosis is mainly caused by attenuated osteogenic differentiation of human bone marrow-derived mesenchymal stem cells (hBMSCs) [3]. Therefore, understanding the regulatory mechanism underlying the differentiation of hBMSCs is crucial for the development of novel treatment approaches to osteoporosis.

Long non-coding RNAs (lncRNAs) are non-coding RNAs, > 200 nt in length [4]. Numerous studies have reported that lncRNAs serve pivotal roles in regulating various biological processes, such as stem cell self-renewal and differentiation [5, 6]. More specifically, several studies have reported that lncRNAs are involved in the pathogenesis of osteoporosis. For instance, lncRNA growth arrest-specific transcript 5 (GAS5) may improve osteoporosis through accelerating MSC osteogenic differentiation via the microRNA (miR)-498/runt-related transcription factor 2 (RUNX2) axis [7]. Additionally, lncRNA nuclear-enriched abundant transcript 1 (NEAT1) could facilitate hBMSC osteogenic differentiation via modulating the miR-29b-3p/bone morphogenetic protein 1 (BMP1) axis [8]. Another study showed that lncRNA MALAT1 could suppress the osteogenic differentiation of MSCs in osteoporotic rats via the MAPK signaling [9]. LncRNA FGD5 antisense RNA 1 (FGD5-AS1) has been reported to enhance osteosarcoma cell proliferation and migration [10]. Recently, Yang et al. demonstrated that FGD5-AS1 expression was downregulated in cartilage tissues of patients with osteoarthritis, and knockdown of FGD5-AS1 inhibited the viability of chondrocytes but promoted apoptosis in osteoarthritis [11]. However, the molecular mechanisms of FGD5-AS1 in hBMSC differentiation remain unclear.

miRNAs are a class of small non-coding RNAs, 20–25 nt in length [12]. They can inversely modulate the expression levels of target genes via binding to the 3′-untranslated region (3′-UTR) of mRNA and further degrade target mRNAs or inhibit mRNA translation [13, 14]. Emerging evidence has suggested that miRNAs can modulate osteogenic differentiation and bone formation [15, 16]. The suppressive effect of miR-506-3p has been verified in several types of cancer [17, 18]. Moreover, miR-506-3p could inhibit osteosarcoma cell proliferation and metastasis by regulating RAB3D [19]. However, its function and regulatory mechanism in osteogenic differentiation have not been previously reported. BMP7, also known as osteogenic protein 1 (OP-1), is a member of the BMP family, which plays a crucial role in osteogenesis and chondrogenesis [20, 21]. A previous study revealed that miR-506-3p downregulated BMP7 to modulate osteoblast viability, differentiation, and migration [22]. However, the specific biological role of the miR-506-3p/BMP7 axis in osteoporosis remains unknown.

The present study aimed to investigate the effect of the FGD5-AS1/miR-506-3p/BMP7 axis on the osteogenic differentiation of BMSCs.

## Materials and methods

### Clinical samples

Bone tissues were collected from patients with osteoporosis (*n* = 17; female, 8; male, 9; age, 51–75 years) and healthy controls (*n* = 17; female, 8; male, 9; age, 50–74 years) at Changzhou Hospital of Traditional Chinese Medicine. The inclusion criteria for patients were as follows: (i) patients provided informed consent and (ii) patients were diagnosed with osteoporosis. Patients with complications due to other diseases, such as chronic inflammatory diseases (such as inflammatory bowel disease and rheumatoid arthritis), were excluded. The present study was approved by the Ethics Committee of Changzhou Hospital of Traditional Chinese Medicine (approval no. CTC20191103A07). Written informed consent was obtained from all patients prior to study enrollment.

### Cell culture

hBMSCs were purchased from the American Type Culture Collection (Catalog number: PCS-500-012) and cultured in DMEM (HyClone; GE Healthcare Life Sciences) supplemented with 10% FBS (Gibco; Thermo Fisher Scientific, Inc.), 100 mg/l streptomycin, and 100 U/l penicillin at 37 °C in a humidified 5% CO_2_ atmosphere. To promote osteogenic differentiation, hBMSCs were maintained in osteogenic medium (OM) with dexamethasone (100 nM) and β-glycerophosphate (2 mM) for 14 days. The medium was replaced every 3 days.

### Cell transfection

Short hairpin RNAs (shRNAs) targeting FGD5-AS1 (shFGD5-AS1) or BMP7 (shBMP7), scramble shRNAs (shNC), miR-506-3p mimic (5′-CAAAGU GCUUACAGUGCAGGUAG-3′), mimics NC (5′-GUCCUGAGAAGGCUAGCA UAGAU-3′), miR-506-3p inhibitor (5′-CUACCUGCACUGUAAGCACUUUG-3′), and inhibitor NC (5′-CUAUGCUAGCCUUCUCAGGACUU-3′) were designed and synthesized by Shanghai GenePharma Co., Ltd., and were transfected into hBMSCs using Lipofectamine® 3000 (Invitrogen; Thermo Fisher Scientific, Inc.). Following transfection for 48 h, the cells were used for subsequent experiments.

### RT-qPCR assay

Total RNA was extracted from hBMSCs using TRIzol® Reagent (Invitrogen; Thermo Fisher Scientific, Inc.) and was reverse-transcribed into cDNA with the PrimeScript RT Reagent Kit (Takara Biotechnology Co., Ltd.). Subsequently, qPCR was carried out on the ABI 7500 Fast Real-Time PCR system (Applied Biosystems; Thermo Fisher Scientific, Inc.) using the SYBR Green Technology (Takara Biotechnology Co., Ltd.). GAPDH and U6 served as internal controls. The 2^−ΔΔCq^ method [23] was used to quantify the relative gene expression levels. The primer sequences were listed as follows: FGD5-AS1, forward 5′-AGTTTCTCTCTAGATT-GCCTT-3′ and reverse 5′-ATTGACATGTTAGTGCCCTT-3′; miR-506-3p, forward 5′-ACACTCATAAGGCACCCTTC-3′ and reverse 5′-TCTACTCAGAAGGGGAGTAC-3′; BMP7, forward 5′-GCAGCACAATTTG GGAA-3′ and reverse 5′-ACAGGTGTTTCGAGAACTGGC-3′; GAPDH, forward 5′-AACGTGTCAGTGGTGGACCTG-3′ and reverse 5′-AGTGGGTGTCGCTGTTGAAGT-3′; and U6, forward 5′-CTCGCTTCGGCAGCACA-3′ and reverse 5′-AACGCTTCACGAATTTGCGT-3′.

### Cell Counting Kit-8 (CCK-8) assay

Cell viability was assessed using a CCK-8 assay (Dojindo Molecular Technologies, Inc.). Briefly, hBMSCs were seeded into a 96-well plate at a density of 5 × 10^3^ cells/well. Subsequently, 10 μl of the CCK-8 solution was added to each well, and cells were incubated at 37 °C for 2 h. The absorbance at 450 nm was used to evaluate the cell proliferation ability using a microplate reader (Bio-Rad Laboratories, Inc.).

### Alkaline phosphatase (ALP) staining and activity assay

ALP activity was assessed using an ALP activity kit (Beyotime) according to the manufacturer’s protocol. The absorbance at a wavelength of 450 nm was measured using a microplate reader (Bio-Tek Instruments, Inc.). For ALP staining, the cells were washed three times with PBS and fixed with 4% paraformaldehyde for 30 min at room temperature. The ALP stain was performed with a BCIP/NBT ALP color development kit (Beyotime).

### Alizarin Red S (ARS) staining

hBMSCs were fixed with 4% paraformaldehyde and stained with 2% ARS (Sigma-Aldrich) for 15 min. Subsequently, cells were washed with PBS and observed under a light microscope (Leica).

### Western blotting

Total proteins were extracted from hBMSCs using a RIPA lysis buffer (Sigma-Aldrich; Merck KGaA) containing protease inhibitors, and the protein concentration was measured by a Pierce BCA Protein Assay Kit (Thermo Fisher Scientific, Inc.). Subsequently, the total protein extracts were separated by 10% SDS-PAGE and transferred onto the PVDF membranes. Following blocking with 5% skimmed milk at 37 °C for 1 h, the membranes were incubated with primary antibodies against OCN (1:1000; Abcam), OPN (1:1000; Abcam), OSX (1:1000; Abcam), RUNX2 (1:1000; Abcam), and GAPDH (1:1000; Abcam) at 4 °C overnight. Then, the membranes were incubated with HRP-conjugated secondary antibodies for 2 h. The bands were visualized using an ECL detection reagent (Invitrogen; Thermo Fisher Scientific, Inc.) and analyzed by the ImageJ software (National Institutes of Health).

### RNA immunoprecipitation (RIP) assay

RIP assay was performed utilizing the EZ-Magna RIP kit (EMD Millipore). Briefly, hBMSCs were lysed in lysis buffer, and cell lysates were incubated with magnetic beads conjugated with anti-Ago2 or anti-IgG antibodies overnight at 4 °C. The samples were then treated with proteinase K. Following washing, the retrieved RNA was analyzed by RT-qPCR assay.

### Bioinformatics analysis and luciferase reporter assay

StarBase v2.0 (http://starbase.sysu.edu.cn/) was used to predict the binding sites between miR-506-3p and FGD5-AS1 or BMP7. The 3′-UTR of FGD5-AS1-wild-type (WT), FGD5-AS1-mutant (MUT), BMP7-WT, and BMP7-MUT were sub-cloned into pmirGLO luciferase vectors (Promega Corporation). Subsequently, hBMSCs were co-transfected with the above plasmids and miR-506-3p or NC mimics using Lipofectamine™ 3000 (Invitrogen; Thermo Fisher Scientific, Inc.). Following transfection for 48 h, hBMSCs were harvested, and the luciferase activity was detected using a Dual-Luciferase Reporter Assay System (Promega Corporation). Firefly luciferase activity was normalized to *Renilla* (Promega Corporation) luciferase gene activity.

### Statistical analysis

Data are expressed as the mean ± SD. All statistical analyses were carried out using the SPSS 21.0 software (IBM Corp.). Statistical differences between the two groups were determined by Student’s *t*-test, while those among multiple groups with one-way ANOVA were followed by Tukey’s post hoc test. *P* < 0.05 was considered to indicate a statistically significant difference.

## Results

### Levels of FGD5-AS1, miR-506-3p, and BMP7 after osteogenic differentiation of BMSCs

**Fig. 1:**
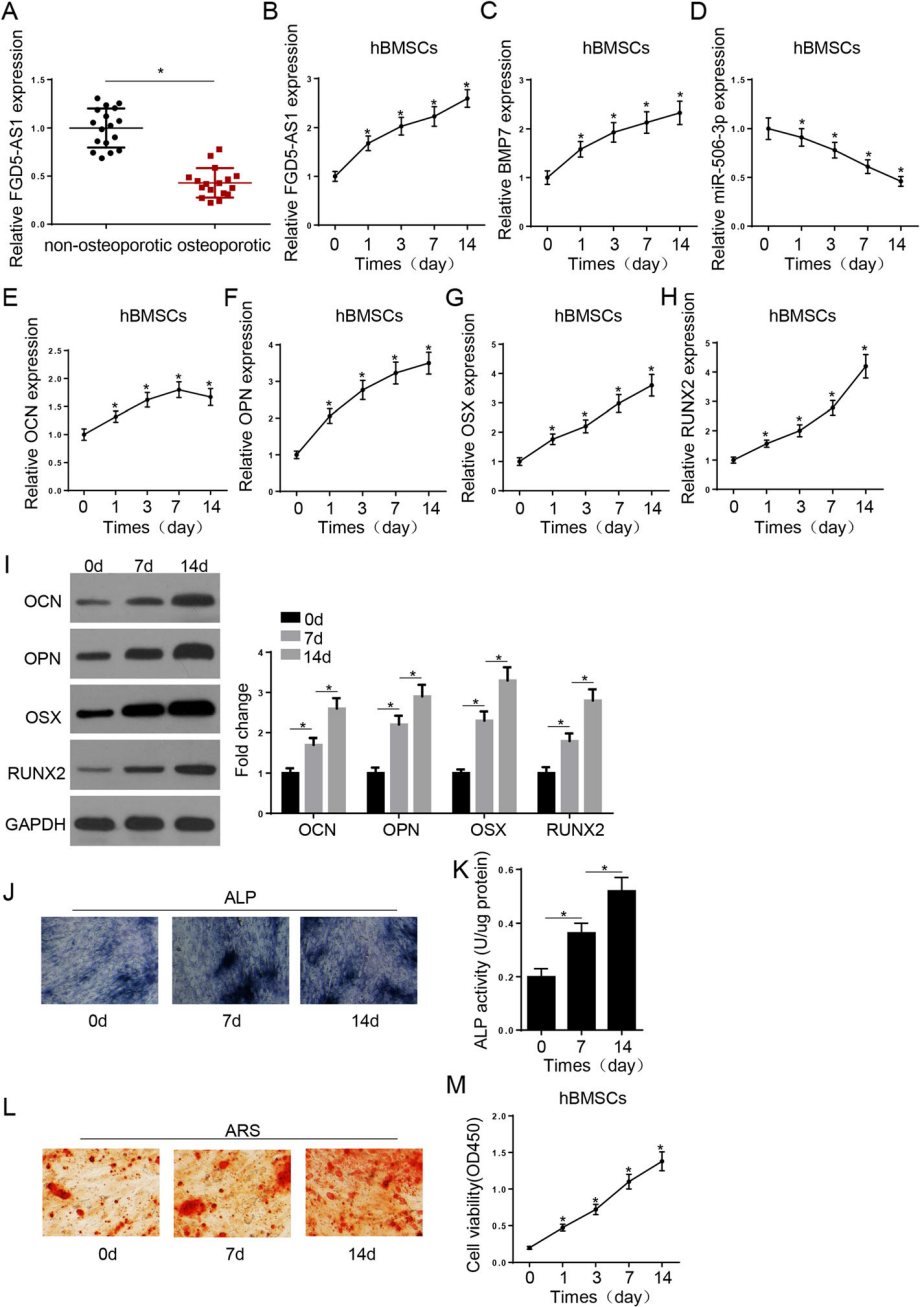
Levels of FGD5-AS1, miR-506-3p, and BMP7 after osteogenic differentiation of BMSCs. **A** RT-qPCR was used to detect the expression of FGD5-AS1 in bone tissues from patients with osteoporosis and healthy individuals. **B**–**H** RT-qPCR analysis was used to detect the expression of FGD5-AS1, miR-506-3p, BMP7, and osteogenesis-related genes (RUNX2, OCN, OSX, and OPN) in hBMSCs treated with OM for 14 days. **I** Western blotting assay showed the protein levels of osteogenesis-related genes in hBMSCs treated with osteogenic medium (OM) for 14 days. **J**, **K** ALP activity and ALP staining assays revealed the ALP activity of hBMSCs treated with OM for 14 days. **K** Alizarin Red staining showed the cell mineralization ability of hBMSCs treated with OM for 14 days. **M** CCK-8 assay showed the viability of hBMSCs during the process of osteogenic differentiation. *P < 0.05

RT-qPCR assay results demonstrated that the FGD5-AS1 expression levels were significantly higher in non-osteoporotic tissues compared with those in tissues from patients with osteoporosis (Fig. 1A). For the induction of osteogenic differentiation, hBMSCs were cultured in OM for 14 days. RT-qPCR indicated that FGD5-AS1 and BMP7 were upregulated from the first day of the induction of hBMSC osteogenic differentiation, whereas miR-506-3p was downregulated after induction of osteogenic differentiation (Fig. 1B–D). Moreover, RT-qPCR analysis revealed that the expression of the osteogenesis-related genes OCN, OPN, OSX, and RUNX2 was notably upregulated (Fig. 1E–H). Consistently, western blot analysis showed that the protein levels of RUNX2, OCN, OSX, and OPN were increased following the culture of hBMSCs in OM (Fig. 1I). ALP staining, ALP activity, and ARS staining assays also showed that the osteogenic differentiation of hBMSCs in OM enhanced ALP staining, ALP activity, and mineralization ability of BMSCs (Fig. 1J–L). Furthermore, the CCK-8 assay demonstrated that hBMSC viability gradually increased during the osteogenic differentiation process (Fig. 1M). Collectively, these findings indicated that FGD5-AS1 could regulate the osteogenic differentiation of hBMSCs.

### FGD5-AS1 knockdown attenuates the osteogenic differentiation of hBMSCs

**Fig. 2:**
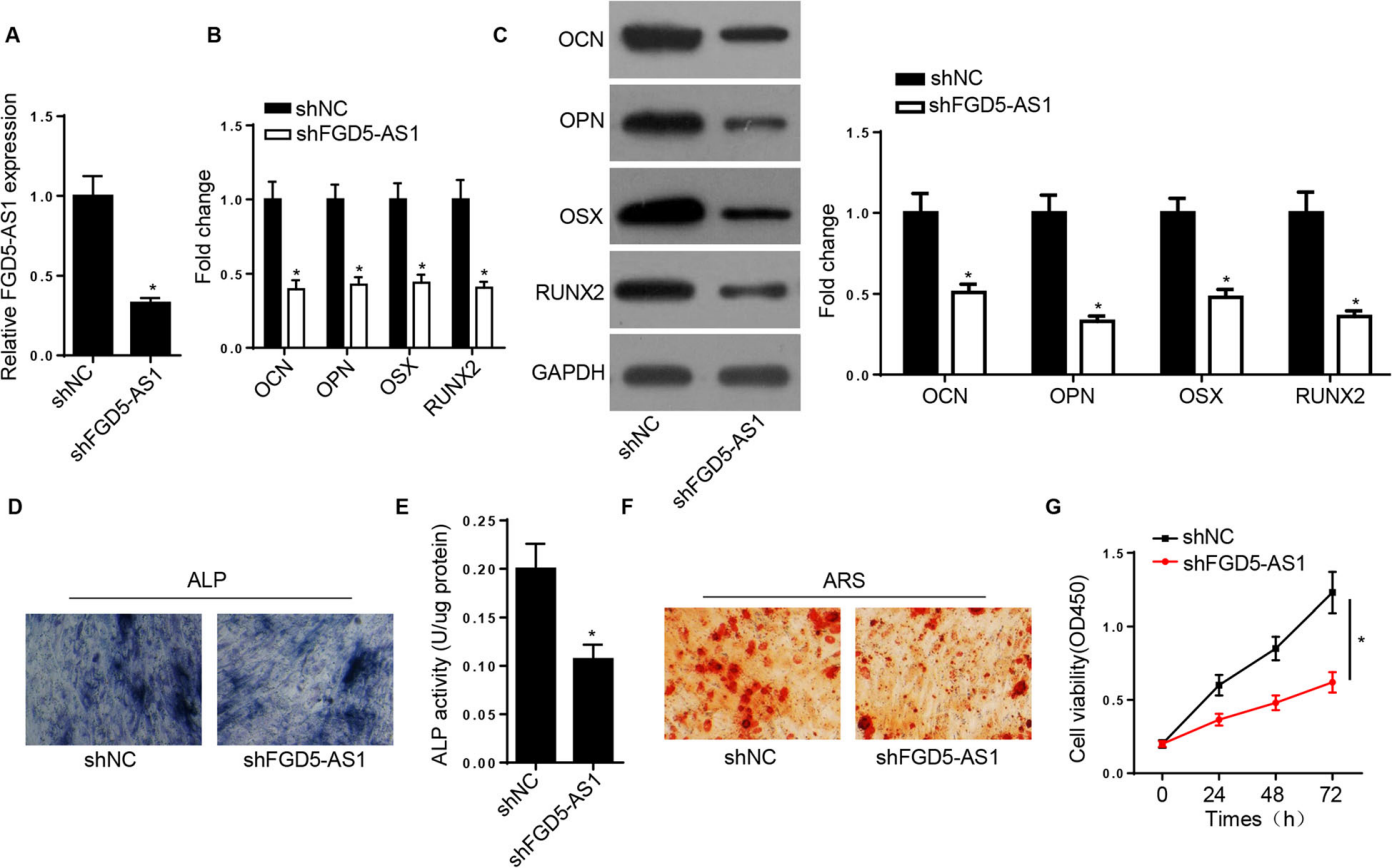
Downregulated FGD5-AS1 suppresses the osteogenesis of hBMSCs. **A** RT-qPCR was used to determine the FGD5-AS1 expression in hBMSCs transfected with shFGD5-AS1 or shNC. **B**, **C** RT-qPCR and western blotting assays showed the protein levels of osteogenic markers in hBMSCs transfected with shFGD5-AS1 or shNC. **D**, **E** ALP activity and ALP staining assays exhibited the ALP activity of hBMSCs transfected with shFGD5-AS1 or shNC. **F** Alizarin Red staining showed the cell mineralization ability of hBMSCs transfected with shFGD5-AS1 or shNC. **G** CCK-8 assay showed the viability of hBMSCs transfected with shFGD5-AS1 or shNC. *P < 0.05

To elucidate the effect of FGD5-AS1 on the osteogenic differentiation of hBMSCs, cells were transfected with shFGD5-AS1 to effectively silence the expression of FGD5-AS1 (Fig. 2A). In addition, the mRNA and protein expression levels of the osteogenic markers, OCN, OPN, OSX, and RUNX2, were reduced after FGD5-AS1 silencing (Fig. 2B, C). Furthermore, FGD5-AS1 depletion reduced the ALP activity and inhibited the mineralization ability in hBMSCs (Fig. 2D–F). Finally, the CCK-8 assay revealed that the hBMSC viability was attenuated by FGD5-AS1 silencing (Fig. 2G). The aforementioned data suggested that FGD5-AS1 knockdown could inhibit the osteogenic differentiation and viability of hBMSCs.

### miR-506-3p is directly targeted by FGD5-AS1

**Fig. 3:**
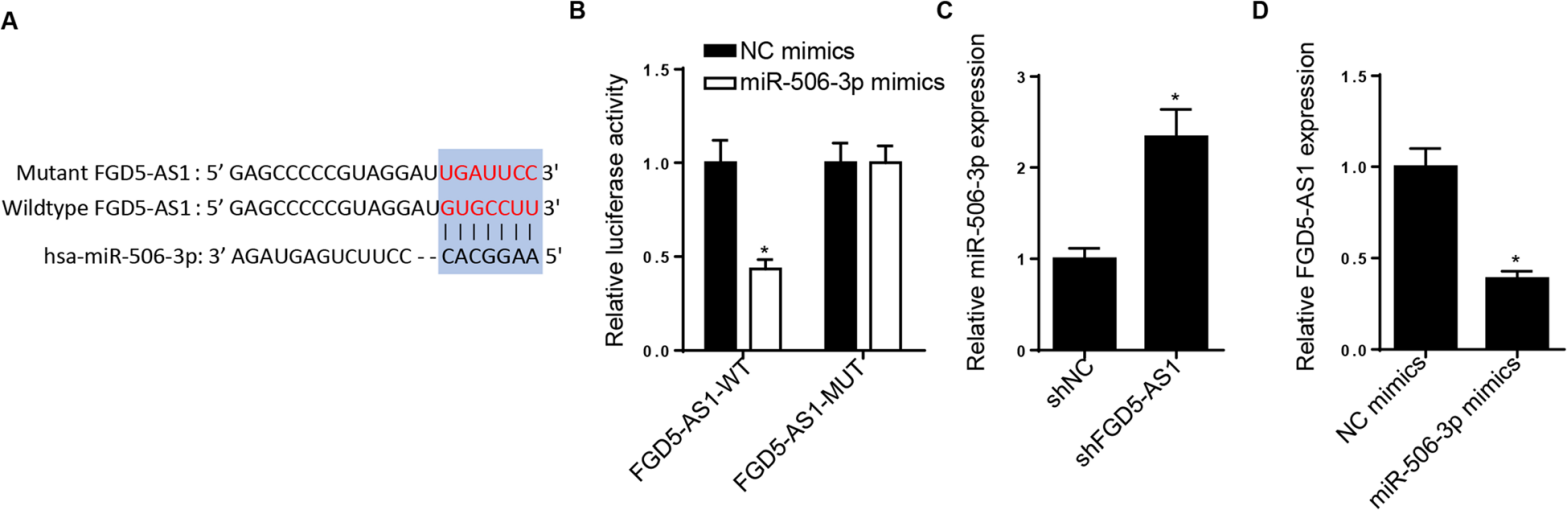
miR-506-3p is a direct target of FGD5-AS1. **A** Binding sequences between FGD5-AS1 and miR-506-3p were predicted by the starBase website. **B** Dual-luciferase reporter assay showed the luciferase activity of FGD5-AS1-WT or FGD5-AS1-MUT in hBMSCs transfected with NC mimics or miR-506-3p mimics. **C** RT-qPCR analysis was used to detect the expression of miR-506-3p in hBMSCs transfected with shFGD5-AS1. **D** RT-qPCR analysis showed the FGD5-AS1 expression in hBMSCs transfected with miR-506-3p mimics. *P < 0.05

Bioinformatics analysis, using the starBase website, predicted that miR-506-3p could be directly targeted by FGD5-AS1 (Fig. 3A). This finding was verified by a dual-luciferase activity reporter assay, and the results demonstrated that miR-506-3p overexpression decreased the luciferase activity of FGD5-AS1-WT, while no changes were observed in cells transfected with FGD5-AS1-MUT (Fig. 3B). In addition, RT-qPCR analysis demonstrated that FGD5-AS1 knockdown increased the miR-506-3p expression (Fig. 3C). Finally, the expression levels of FGD5-AS1 were decreased following transfection of hBMSCs with miR-506-3p mimics (Fig. 3D). Taken together, these findings indicated that FGD5-AS1 could directly bind with miR-506-3p.

### miR-506-3p silencing rescues the suppressive effect of FGD5-AS1 downregulation on the osteogenic differentiation of hBMSCs

**Fig. 4:**
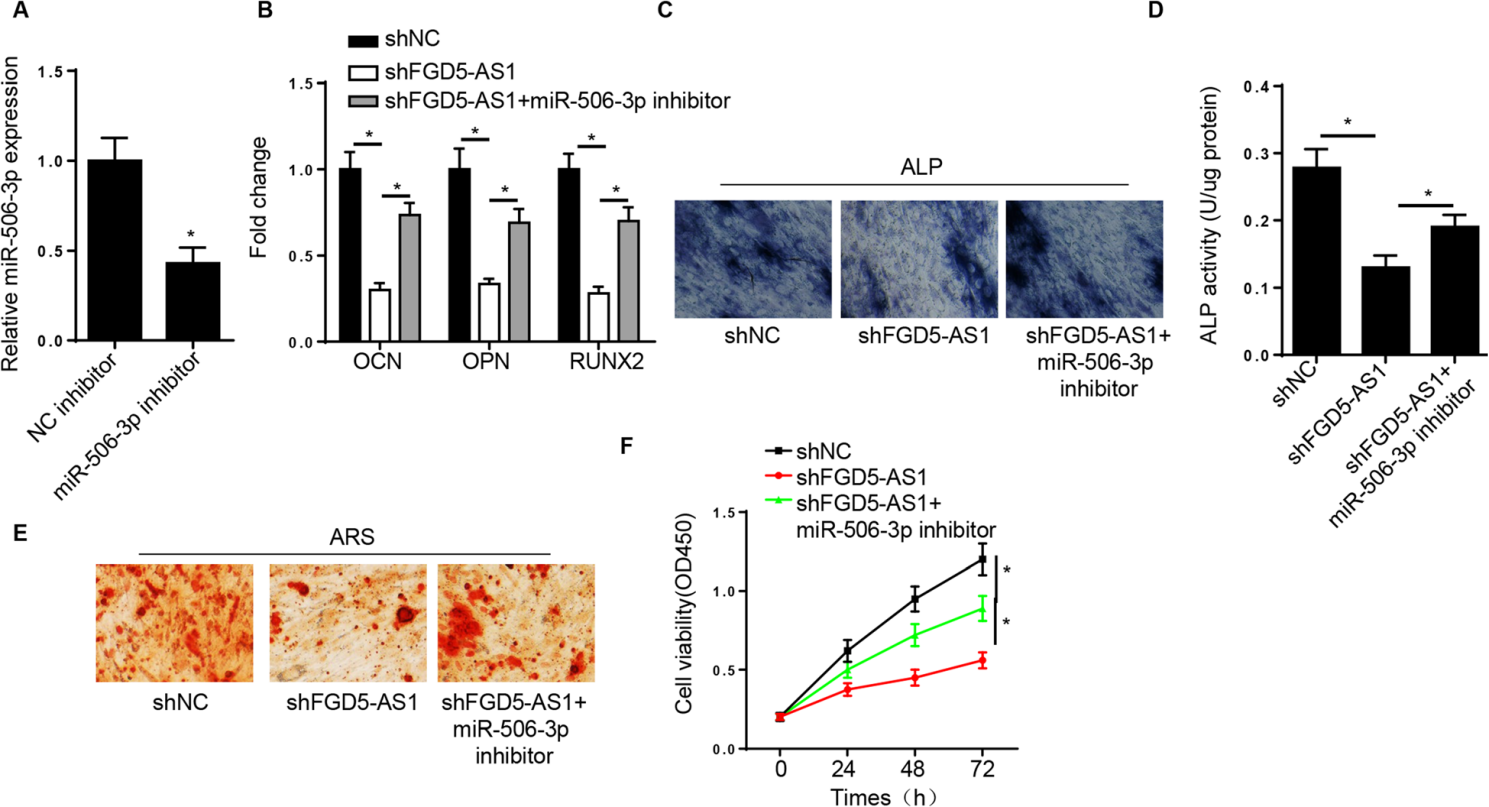
Decreased miR-506-3p rescues the suppressive effect of FGD5-AS1 depletion on the osteogenesis of hBMSCs. **A** RT-qPCR analysis showed the miR-506-3p levels in hBMSCs transfected with NC inhibitor and miR-506-3p inhibitor. **B** RT-qPCR analysis showed the levels of osteogenic markers in hBMSCs transfected with shNC, shFGD5-AS1, and shFGD5-AS1+miR-506-3p inhibitor. **C**, **D** ALP staining and ALP activity assays showed the ALP activity of hBMSCs transfected with shNC, shFGD5-AS1, and shFGD5-AS1+miR-506-3p inhibitor. **E** Alizarin Red staining showed the cell mineralization ability of hBMSCs transfected with shNC, shFGD5-AS1, and shFGD5-AS1+miR-506-3p inhibitor. **F** CCK-8 assay determined the viability of hBMSCs transfected with shNC, shFGD5-AS1, and shFGD5-AS1+miR-506-3p inhibitor. *P < 0.05

Subsequently, we investigated whether FGD5-AS1 could regulate osteogenic differentiation via targeting miR-506-3p. The transfection efficiency of the miR-506-3p inhibitor was confirmed by RT-qPCR (Fig. 4A). As shown in Fig. 4B, FGD5-AS1 knockdown reduced the expression of OCN, OPN, and RUNX2 in hBMSCs, while this effect was reversed after transfection of hBMSCs with miR-506-3p inhibitor. In addition, miR-506-3p silencing rescued the reduced ALP activity and mineralized nodules mediated by FGD5-AS1 knockdown in hBMSCs (Fig. 4C–E). Additionally, the CCK-8 assay revealed that FGD5-AS1 knockdown attenuated the viability of hBMSCs, which was further promoted by miR-506-3p downregulation (Fig. 4F). These data indicated that miR-506-3p silencing could rescue the suppressive effect of FGD5-AS1 downregulation on the osteogenic differentiation of hBMSCs.

### FGD5-AS1 upregulates BMP7 expression via binding with miR-506-3p

**Fig. 5:**
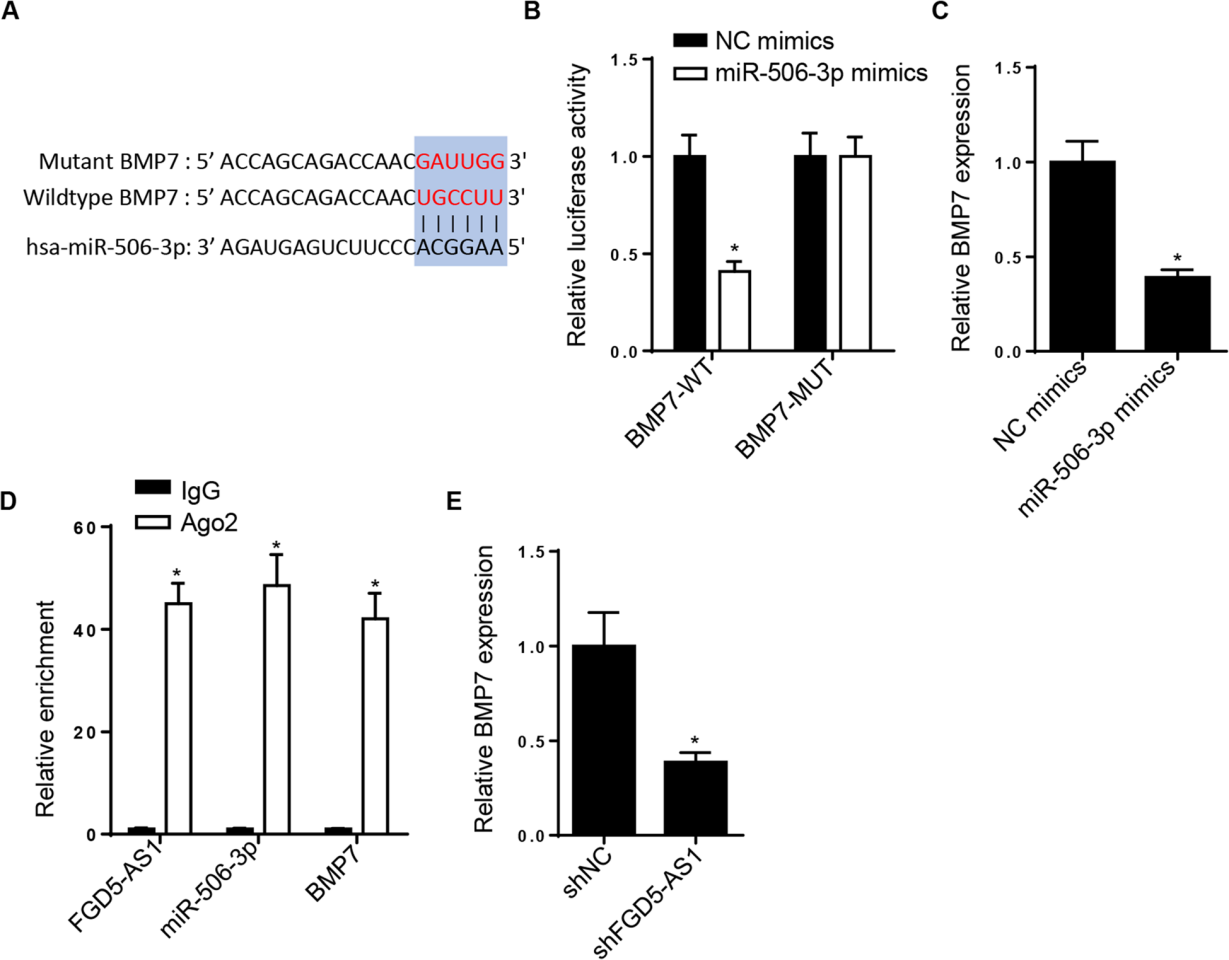
FGD5-AS1 upregulates BMP7 via binding to miR-506-3p. **A** Binding sequences between BMP7 and miR-506-3p were predicted by the starBase website. **B** Dual-luciferase reporter assay showed the luciferase activity of BMP7-WT or BMP7-MUT in hBMSCs transfected with NC mimics or miR-506-3p mimics. **C** RT-qPCR analysis was used to detect the expression of BMP7 in hBMSCs transfected with NC mimics or miR-506-3p mimics. **D** RIP assay showed the enrichment of FGD5-AS1, miR-506-3p, and BMP7 in the anti-Ago2 group compared with the anti-IgG group. **E** RT-qPCR analysis showed the expression of BMP7 in hBMSCs transfected with shNC and shFGD5-AS1

The starBase website was utilized to predict the potential binding sites between miR-506-3p and BMP7. The putative binding sequences of miR-506-3p and BMP7 are shown in Fig. 5A. Subsequently, the luciferase reporter assay verified that miR-506-3p overexpression obviously decreased the luciferase activity of BMP7-WT. However, no significant changes were obtained in cells transfected with BMP7-MUT (Fig. 5B). Additionally, RT-qPCR analysis showed that miR-506-3p mimics markedly downregulated BMP7 (Fig. 5C). Furthermore, RIP assays demonstrated that the expression levels of FGD5-AS1, miR-506-3p, and BMP7 were enriched in the anti-Ago2 group (Fig. 5D). Additionally, FGD5-AS1 depletion decreased the expression levels of BMP7 (Fig. 5E). Overall, these results indicated that FGD5-AS1 could modulate the BMP7 expression via sponging miR-506-3p.

### Silencing of BMP7 abrogates the promoting effect of the miR-506-3p inhibitor on the osteogenic differentiation of hBMSCs

**Fig. 6:**
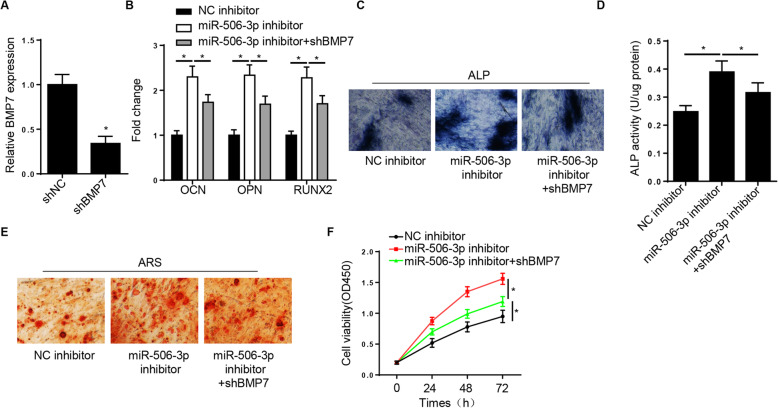
Silence of BMP7 abrogates the promotion effect of the miR-506-3p inhibitor on osteogenesis of hBMSCs. **A** RT-qPCR analysis showed the BMP7 expression in hBMSCs transfected with shNC or shBMP7. **B** RT-qPCR analysis showed the OCN, OPN, and RUNX2 levels in hBMSCs transfected with NC inhibitor, miR-506-3p inhibitor, and miR-506-3p inhibitor+shBMP7. **C**–**F** ALP activity, ALP staining, ARS staining, and CCK-8 assays showed the ALP activity and viability of hBMSCs transfected with NC inhibitor, miR-506-3p inhibitor, and miR-506-3p inhibitor+shBMP7. *P < 0.05

Subsequently, to investigate the role of BMP7 in promoting the FGD5-AS1-mediated osteogenic differentiation, shRNAs targeting BMP7 were synthesized to suppress the BMP7 expression (Fig. 6A). RT-qPCR analysis showed that miR-506-3p silencing increased the expression levels of OCN, OPN, and RUNX2 in hBMSCs, while BMP7 knockdown reversed this effect (Fig. 6B). In addition, BMP7 downregulation partially reversed the enhanced ALP activity, mineralized nodules, and cell viability caused by miR-506-3p inhibition (Fig. 6C–F). Overall, these results suggested that FGD5-AS1 could enhance osteogenic differentiation via regulating the miR-506-3p/BMP7 axis.

## Discussion

Osteoporosis is an age-associated skeletal disease characterized by decreased bone mass and increased bone fragility and fracture risk [24]. An increasing number of individuals are currently suffering from osteoporosis [25]. Although progress has been made in the treatment of osteoporosis, the mechanisms involved in this disease remain unclear [26]. Previous studies have shown that hBMSCs have the potential of multidirectional differentiation and can be transformed into osteoblasts in response to appropriate stimuli [27]. Therefore, it is crucial to elucidate the regulatory mechanism underlying the osteogenic differentiation of hBMSCs.

Emerging evidence has suggested that lncRNAs serve vital roles in the progression of osteoporosis via regulating osteogenic differentiation. For example, a study demonstrated that lncRNA MSC-AS1 could inhibit the development of osteoporosis by promoting the osteogenic differentiation of BMSCs via regulating the miR-140-5p/BMP2 axis [28]. HOTAIR was shown to suppress the osteogenic differentiation of BMSCs via modulating the Wnt/β-catenin pathway [29]. Another study reported that lncRNA GAS5 accelerated osteogenic differentiation of BMSCs via targeting miR-135a-5p to regulate FOXO1 [30]. It has been reported that lncRNA FGD5 antisense RNA1 (FGD5-AS1) serves a crucial role in the progression of several types of malignant tumors [31, 32]. However, its effect on the osteogenic differentiation of hBMSCs remains unclear. Herein, the expression of FGD5-AS1 was upregulated during osteogenic differentiation. Furthermore, FGD5-AS1 silencing attenuated the osteogenic differentiation processes of hBMSCs.

Emerging evidence has suggested that lncRNAs act as sponges for miRNAs to modulate the expression of their target mRNAs [33]. Previous studies have indicated that miRNA may play a vital role in regulating the pathogenesis of various diseases [34–36]. Herein, we identified that miR-506-3p was a downstream target of FGD5-AS1. Moreover, miR-506-3p was upregulated by FGD5-AS1 depletion, while miR-506-3p overexpression reduced the expression of FGD5-AS1. Functional assays revealed that miR-506-3p downregulation reversed the suppressive effect of FGD5-AS1 silencing on ALP activity and viability of hBMSCs. Subsequently, BMP7 was identified as a direct target of miR-506-3p. Bone morphogenetic proteins (BMPs), as members of the transforming growth factor-b (TGF-b) superfamily, play a vital role in inducing bone formation [37]. BMP-7 has been reported to stimulate a chondrogenic phenotype in adipose tissue-derived stem cells and enhance the chondrogenesis of synovial MSC when combined with TGF-b1 [38]. Moreover, Liu et al. reported that miR-542-3p modulated the osteogenic differentiation of vascular smooth muscle cells via targeting BMP7 [39]. Another study showed that BMP7 could promote osteogenic differentiation of human periosteal cells in vitro [40]. The results of the present study revealed that deletion of miR-506-3p increased ALP activity, mineralized nodules, and viability of hBMSCs, while BMP7 downregulation reversed these effects. The aforementioned findings suggested that FGD5-AS1 could promote the osteogenic differentiation of hBMSCs via the miR-506-3p/BMP7 axis.

## Conclusion

To the best of our knowledge, the present study is the first to investigate the effect of FGD5-AS1 on the progression of osteogenic differentiation. The results demonstrated that FGD5-AS1 could promote the osteogenic differentiation of hBMSCs via regulating the miR-506-3p/BMP7 axis, thus providing novel insights into the mechanisms underlying the osteogenic differentiation of hBMSCs. However, there are several factors affecting osteoporosis, while the present study mainly focused on the effects of osteogenic differentiation on osteoporosis. Therefore, further investigations are needed to uncover the possible involvement of other mechanisms in the regulation of osteoporosis.

## Data Availability

The datasets used and/or analyzed during the current study are available from the corresponding author on reasonable request.

## Abbreviations

hBMSCs: Human bone marrow-derived mesenchymal stem cells
lncRNAs: Long non-coding RNAs
FGD5-AS1: FGD5 antisense RNA 1
ALP: Alkaline phosphatase
CCK-8: Cell Counting Kit-8
BMP7: Bone morphogenetic protein 7
3′-UTR: 3′-Untranslated region
GAS5: Growth arrest-specific transcript 5
RUNX2: Runt-related transcription factor 2
BMP1: Bone morphogenetic protein 1
OP-1: Osteogenic protein 1
BMPs: Bone morphogenetic proteins
TGF-b: Transforming growth factor-b
